# Foldable Perovskite Solar Cells Using Carbon Nanotube‐Embedded Ultrathin Polyimide Conductor

**DOI:** 10.1002/advs.202004092

**Published:** 2021-02-08

**Authors:** Jungjin Yoon, Unsoo Kim, Yongseok Yoo, Junseop Byeon, Seoung‐Ki Lee, Jeong‐Seok Nam, Kyusun Kim, Qiang Zhang, Esko I. Kauppinen, Shigeo Maruyama, Phillip Lee, Il Jeon

**Affiliations:** ^1^ Photo‐Electronic Hybrids Research Center, National Agenda Research Division Korea Institute of Science and Technology (KIST) Seoul 02792 Republic of Korea; ^2^ Department of Materials Science & Engineering Pennsylvania State University University Park PA 16802 USA; ^3^ Department of Mechanical Engineering Seoul National University Seoul 08826 Republic of Korea; ^4^ Global Frontier Center for Multiscale Energy Systems Seoul National University Seoul 08826 Republic of Korea; ^5^ Institute of Advanced Composite Materials Korea Institute of Science and Technology (KIST) Wanju 55324 Republic of Korea; ^6^ Department of Chemistry Education, Graduate School of Chemical Materials, Institute for Plastic Information and Energy Materials, Sustainable Utilization of Photovoltaic Energy Research Center (ERC) Pusan National University Busan 46241 Republic of Korea; ^7^ Department of Applied Physics Aalto University School of Science Aalto FI‐00076 Finland; ^8^ Department of Mechanical Engineering, School of Engineering The University of Tokyo Tokyo 113‐8656 Japan

**Keywords:** carbon nanotube and polyimide matrix, flexible solar cells, flexible transparent conductors, foldable electronics, single‐walled carbon nanotubes

## Abstract

Recently, foldable electronics technology has become the focus of both academic and industrial research. The foldable device technology is distinct from flexible technology, as foldable devices have to withstand severe mechanical stresses such as those caused by an extremely small bending radius of 0.5 mm. To realize foldable devices, transparent conductors must exhibit outstanding mechanical resilience, for which they must be micrometer‐thin, and the conducting material must be embedded into a substrate. Here, single‐walled carbon nanotubes (CNTs)–polyimide (PI) composite film with a thickness of 7 µm is synthesized and used as a foldable transparent conductor in perovskite solar cells (PSCs). During the high‐temperature curing of the CNTs‐embedded PI conductor, the CNTs are stably and strongly *p*‐doped using MoO*_x_*, resulting in enhanced conductivity and hole transportability. The ultrathin foldable transparent conductor exhibits a sheet resistance of 82 Ω sq.^−1^ and transmittance of 80% at 700 nm, with a maximum‐power‐point‐tracking‐output of 15.2% when made into a foldable solar cell. The foldable solar cells can withstand more than 10 000 folding cycles with a folding radius of 0.5 mm. Such mechanically resilient PSCs are unprecedented; further, they exhibit the best performance among the carbon‐nanotube‐transparent‐electrode‐based flexible solar cells.

## Graphical abstract

Ultrathin MoO*_x_*‐doped carbon nanotube‐embedded polyimide films are fabricated as transparent substrates for foldable perovskite solar cells. The carbon nanotube‐polyimide matrix prevents the peeling of carbon electrode from the substrate and facilitates anaerobic thermal *p*‐doping using MoO*_x_*. The transparent conductors exhibit state‐of‐the‐art mechanical stability because of their ultra‐thinness and record‐high efficiency and outstanding durability.

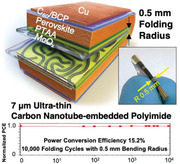

In recent years, the growing demand for deformable electronics has spurred extensive research in this field.^[^ ^1^, ^2^, ^3^ ^]^ Consequently, the research community has witnessed numerous publications on smart clothing,^[^ ^4^, ^5^, ^6^ ^]^ and artificial optoelectrical skins.^[^ ^7^, ^8^, ^9^ ^]^ This is now a mature field, with foldable cell phones having already been spun out into the commercial market.^[^ ^10^ ^]^ Energy devices, such as photovoltaic devices are following suit in the form of hair‐wrapping‐flexible organic solar cells^[^ ^11^ ^]^ and perovskite solar cells (PSCs) that can be crumpled.^[^ ^12^, ^13^ ^]^ In deformable electronics technology, transparent conductors and substrates play the most crucial role, governing the entire mechanical flexibility of the device. This is because while thin‐film electronics demonstrate enough flexibility, conventional metal oxide transparent conductors such as indium tin oxide (ITO) and rigid glass substrates limit device flexibility. Much effort has been devoted to the replacement of ITO with metal nanowire‐based composites,^[^ ^14^, ^15^ ^]^ polymer‐based electrodes,^[^ ^12^ ^]^ and multi‐layered conductors.^[^ ^16^, ^17^, ^18^ ^]^

Over the last 20 years, carbon nanotubes (CNTs) have generated significant excitement among researchers owing to their applicability in electronics.^[^ ^19^, ^20^ ^]^ In addition to high conductivity, CNTs demonstrate high transparency with remarkable mechanical resilience. They are thus considered a promising alternative to conventional electrodes, namely, ITO^[^ ^21^, ^22^, ^23^, ^24^, ^25^, ^26^ ^]^ and metals.^[^ ^26^, ^27^, ^28^ ^]^ While the applicability of CNTs to flexible devices has been extensively studied,^[^ ^21^, ^29^, ^30^ ^]^ the application of CNTs in foldable devices has not yet been reported. This is due to the more demanding requirements of the foldable device technology. Because of the severe bending conditions (bending radius of 0.5 mm) of foldable devices, CNTs to be applied in foldable devices require not only high mechanical stability but also a high adhesion strength for the substrates. Similar to hydrophobic carbon electrodes, CNTs also peel off from devices under severe stresses. To avoid such a problem, a CNT–polymer matrix with excellent durability and mechanical robustness has been reported as a conducting substrate.^[^ ^31^, ^32^ ^]^ However, the application of these systems in foldable devices has not yet been reported, and those used in flexible devices demonstrate mediocre optoelectrical performance.

The performance of carbon electrodes is directly related to chemical doping. The DC‐to‐optical conductivity ratio of pristine carbon electrodes is not comparable to that of ITO, and a high ratio can be achieved only through chemical doping. While strong acids such as HNO_3_^[^ ^33^ ^]^ and CF₃SO₃H,^[^ ^34^ ^]^ have been preferred *p*‐dopant, their corrosiveness and instability raise concerns. Furthermore, solution‐based chemical doping is unviable for polymer‐CNT matrices as contact between the acid and CNTs, which is required for *p*‐doping, is hindered by the polymer encapsulation of CNTs. However, MoO*_x_* doping proposed by Bao and coworkers,^[^ ^35^ ^]^ overcomes the abovementioned problems. MoO_3_ is safe to handle and displays excellent doping durability. Nevertheless, to induce a strong doping effect using MoO_3_, the thermal annealing of MoO_3_ next to CNTs at a high temperature of approximately 300 °C under anaerobic conductions is necessary. In this regard, the CNT–polymer matrix is the perfect candidate, as it can be formed after the direct deposition of MoO_3_ on CNTs, and polymers with a high glass transition temperature, (*T*_g_) can be used for the CNT–polymer matrix to utilize MoO_3_ as the dopant.

In this study, we prepared a single‐walled carbon nanotube (SWNT)–polyimide (PI) matrix in which a strong *p*‐doping can be induced with MoO_3_ at a high temperature under anaerobic conditions. The resulting SWNT‐embedded PI (SWNT–PI) film displayed an exceptionally smooth morphology and high transparency thanks to the infiltration of the SWNTs into PI, which results in the filling of the network voids. The high *T*_g_ of PI (300 °C) and the effective filling of voids using the SWNTs facilitated effective thermal and anaerobic *p*‐doping using MoO_3_. The sheet resistance (*R*_sheet_) of the SWNT–PI conductor decreased upon doping through the reduction of MoO_3_ to MoO*_x_*, (*x* = 2–3).^[^ ^29^ ^]^ The MoO*_x_*‐doped SWNT–PI conductor is 7 µm in thickness, which enables not only bending but also the “folding” of the devices without any photovoltaic performance drop or decrease in the DC‐to‐optical conductivity ratio. The foldable PSCs fabricated using the ultrathin MoO*_x_*‐doped SWNT–PI film as both the electrode and substrate exhibited a power conversion efficiency (PCE) of 15.2% with an open‐circuit voltage (*V*_OC_) of 1.05 V, short‐circuit current (*J*_SC_) of 19.0 mA cm⁻^2^, and a fill factor (FF) of 76.6%. The devices withstood more than 10 000 “folding” cycles with a 0.5 mm folding radius, whereas the control PSCs on ITO‐coated polyethylene naphthalate (PEN) exhibited severe performance degradation after 1 000 “bending” cycles with 4 mm radius and could not endure even one folding cycle. The extreme folding radius of 0.5 mm presented in this work is by far the most severe condition compared with those reported previously for flexible devices as most of the demonstrated bending radius is 4 mm. To the best of our knowledge, the only electrode that exhibits a comparable folding radius is poly(3,4‐ethylenedioxythiophene) polystyrene sulfonate (PEDOT:PSS). The foldable PSC based on the ultrathin MoO*_x_*‐doped CNT–PI conductor exhibited a record‐high PCE among the CNT‐based flexible PSCs and the highest flexibility (folding radius: 0.5 mm) and mechanical robustness (10 000 folding cycles) among all the flexible PSCs reported thus far.

**Figure** **1** shows the schematic of the fabrication of the novel SWNT‐based ultrathin foldable transparent conductor. First, a thin layer of MoO_3_ was thermally deposited on a quartz substrate (Figure 1a). This is followed by the lamination of an SWNT film (Figure 1b). Immediately after MoO_3_ contacts the SWNT film, charge transfer occurs and MoO_3_ is partially reduced to MoO_3‐_ *_*δ*_*. Hereafter, MoO_3‐_ *_*δ*_* is referred to as MoO*_x_* (*x* = 2 to 3). Next, a PI precursor solution is applied and spin‐coated on the SWNT/MoO*_x_*/quartz substrate (Figure 1c).^[^ ^36^ ^]^ The viscous PI solution percolates through the SWNT network, forming an SWNT–PI nanocomposite. The nanocomposite film is then cured and imidized by heat. During this stage, the electron transfer from the SWNT to MoO*_x_* (*p*‐doping) intensifies as the curing temperature reaches 300 °C and MoO*_x_* is completely screened from oxygen. The cured SWNT–PI film adheres to the quartz via van der Waals forces; therefore, it can be easily removed. Thus, when immersed in deionized (DI) water, the SWNT–PI film peels off without damage (Figure 1d). Subsequently, the film is dried and turned over such that the MoO*_x_* side faces upward, yielding ultrathin, transparent, and foldable SWNT‐embedded PI electrodes (Figure S1, Supporting Information). The electrode is denoted as SWNT–PI (without MoO*_x_*) or MoO*_x_*/SWNT–PI (Figure 1e). The morphology of transparent electrodes is crucial for the solar cell performance.^[^ ^25^ ^]^ Figure 1f–h show the atomic force microscopy (AFM) images of the surface topography of the SWNT–PI conductors with their root mean square (rms) roughness values shown at the bottom of the images. The SWNT–PI films exhibit an extremely low rms roughness of 0.456 nm, indicating extremely smooth surfaces (Figure 1f). The MoO*_x_*/SWNT–PI films with a several‐nanometer‐thick MoO*_x_* layer also exhibited a low rms roughness value of 0.446 nm (Figure 1g). Such flat morphologies were achieved owing to the permeation of the PI precursor into the entangled SWNT network and filling of the voids at the quartz interface (Figure 1c). In the case of the MoO*_x_*/SWNT–PI films with greater MoO*_x_* thicknesses, they likewise give good morphology with rms values less than 0.5 nm (Figure S2, Supporting Information). Contrarily, the SWNT films on PEN substrates exhibited a high rms roughness of 7.040 nm because of the exposed entangled SWNTs (Figure 1h and Figure S3, Supporting Information). Such a high roughness results in a low PCE when assembled into PSCs.^[^ ^21^, ^25^ ^]^ The intrinsically entangled geometry of the SWNT network and the voids render the application of SWNTs as a bottom electrode unbearably challenging. Therefore, our PI embedding technology offers a novel way of producing SWNT‐based transparent conductors with a good morphology, thereby preventing electrical current leakage when used in thin‐film devices.^[^ ^37^, ^38^ ^]^ Figure 1i shows the optical transmittance of the SWNT–PI conductors. The transmittance of the PI film and SWNT–PI conductors are ≈80% and 88% in the wavelength range of 450–800 nm, respectively (Figure S4, Supporting Information). The optical transparency is comparable to those of conventional ITO/PEN substrates,^[^ ^39^ ^]^ and thus the films qualify for the transparent electrodes and the bottom substrates. The PI conductors exhibited optical interference in the long‐wavelength region, which indicates their ultra‐thinness (7 µm) (Figure 1i).^[^ ^40^, ^41^ ^]^ This is confirmed from the PI film thickness measured via optical microscopy (Figure S5, Supporting Information).

**Figure 1:**
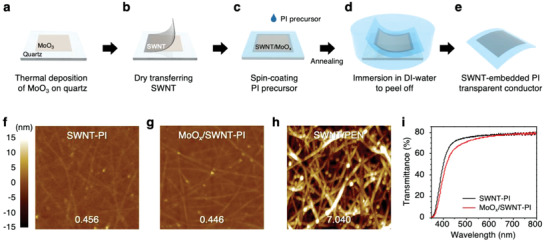
Fabrication of the ultrathin SWNT–PI conductor. a–e) Schematic illustration of the fabrication process of SWNT–PI ultrathin conductors: a) thermal deposition of MoO_3_ on a quartz glass substrate, b) dry transfer of SWNT film, c) spin‐coating of PI precursor and annealing to cure the PI film and *p*‐dope the SWNTs with MoO*_x_*, d) lifting off of the SWNT–PI conductor from the glass substrate, and e) the resulting ultrathin transparent conductor. AFM topography images of f) SWNT–PI, g) MoO*_x_*/SWNT–PI, and h) SWNT/PEN substrates (area: 1.5 × 1.5 µm^2^, rms roughness values are displayed on each image), and i) optical transmittance spectra of the SWNT–PI‐based films.

The *p*‐doping of carbon electrodes is crucial as it increases the DC‐to‐optical conductivity ratio of the carbon electrodes and tunes the Fermi level, resulting in the performance enhancement of optoelectronic devices.^[^ ^20^ ^]^ MoO_3_ on CNTs induces strong and stable *p*‐doping of the CNTs by reducing to MoO*_x_* ^[^ ^29^ ^]^ The creation of oxygen traps through electron extraction from CNTs increases the hole carrier concentration and lowers the Fermi level of the CNTs.^[^ ^29^, ^42^ ^]^ To maximize the doping effect, the MoO_3_‐deposited carbon electrode was annealed at a high temperature of over 300 °C under anaerobic conditions. The curing process of the ultrathin SWNT–PI conductor is illustrated in **Figure** **2**a. The high curing temperature of PI (300 °C) and the protection of MoO_3_ from air during the curing process ensure the successful MoO*_x_* doping of SWNTs, which occurs automatically during the production of the SWNT–PI matrix (Figures 1a and 2a). Besides, since PI has an exceptionally high *T*_g_, only PI is exclusively compatible with this technology. The doping effect was confirmed via the X‐ray photoelectron spectroscopy (XPS) of the films before and after the doping (Figure 2b,c and Figure S6, Supporting Information). Figure 2b shows the Mo 3d spectra of MoO*_x_*/SWNT–PI and MoO*_x_*/SWNT/PEN. Compared with MoO*_x_*/SWNT/PEN, MoO*_x_*/SWNT–PI exhibits a weaker Mo 3d peak as the MoO*_x_*‐deposited SWNTs are embedded in PI. Both MoO*_x_*/SWNT–PI and MoO*_x_*/SWNT/PEN exhibited peaks at 232.6 and 235.8 eV corresponding to the Mo^6+^ and Mo^5+^ binding energies, respectively. However, MoO*_x_*/SWNT–PI shows an additional peak at 229.8 eV corresponding to the binding energy of the Mo^4+^ intermediate. This accounts for the strong MoO*_x_* doping of the MoO*_x_*/SWNT–PI film via the high‐temperature curing process.^[^ ^35^, ^42^, ^43^ ^]^ Figure 2c shows the O 1s XPS spectra of SWNT–PI, MoO*_x_*/SWNT–PI, and MoO*_x_*/SWNT/PEN. The peak at 530 eV corresponds to the oxygen in MoO_3_, whereas the peak between 532 and 533 eV corresponds to the oxygen adsorbed on carbon. The oxygen peak appearing at a higher binding energy for the MoO*_x_*/SWNT–PI films indicates the strong doping effect.^[^ ^35^, ^44^, ^45^ ^]^ The degree of doping can also be determined from the Van Hove transitions corresponding to Vis–IR absorption.^[^ ^34^, ^46^, ^47^, ^48^ ^]^ However, the optical interference in the absorption caused by the PI film hinders the visualization of the transitions for the MoO*_x_*/SWNT–PI films (Figure S7, Supporting Information).

**Figure 2:**
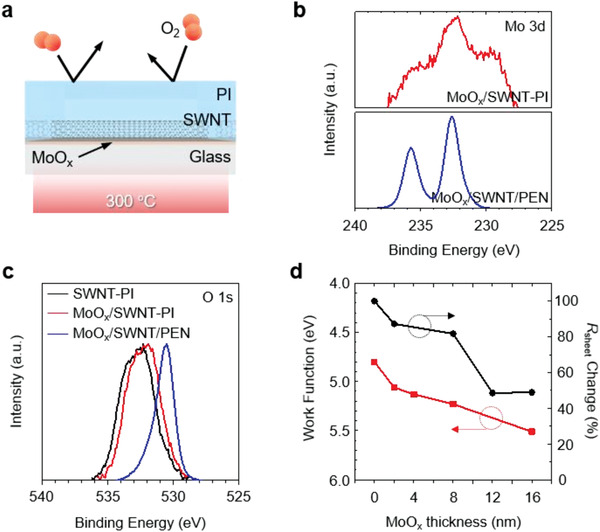
Strong MoO*_x_*‐doping of SWNT electrode. a) Schematic illustration of the annealing mechanism of the SWNT–PI conductor, b) Mo 3d XPS spectra of MoO*_x_*/SWNT–PI and MoO*_x_*/SWNT/PEN, c) O 1s XPS spectra of SWNT–PI, MoO*_x_*/SWNT–PI, and MoO*_x_*/SWNT/PEN, and d) changes in work function and sheet resistance of MoO*_x_*/SWNT–PI as a function of MoO*_x_* thickness.

MoO*_x_* deposited by thermal evaporation under vacuum promotes film growth through the Volmer–Weber nucleation mechanism.^[^ ^49^ ^]^ Such growth promotes island formation when the thickness of the deposited MoO*_x_* is several nanometers. The *R*_sheet_ of SWNT–PI decreased with increasing MoO*_x_* thickness (Figure 2d), indicating stronger *p*‐doping with increasing MoO*_x_* deposition. Further, the decrease in *R*_sheet_ was not observed when a 10‐nm‐thick MoO*_x_* layer was deposited. This indicates the complete coverage of the CNT surface by MoO*_x_*. However, the work function measured by ultraviolet photoelectron spectroscopy (UPS) continuously decreased with an increase in MoO*_x_* thickness above 10 nm (Figure 2d and Figure S8, Supporting Information). Thus, determining the optimal MoO*_x_* thickness is important with regard to achieving a balance between the *R*_sheet_ and energy level alignment for PSC performance.

Foldable PSCs employing the ultrathin SWNT–PI conductor were fabricated with a *p‐i‐n* inverted configuration: SWNT–PI/MoO*_x_*/poly(triarylamine) (PTAA)/perovskite/C_60_/bathocuproine (BCP)/Cu (**Figure** **3**a). The inverted architecture is suitable for transparent CNT applications because CNTs have a work function of approximately 5 eV (Figure 2d and Figure S8, Supporting Information).^[^ ^33^, ^50^, ^51^ ^]^ As aforementioned, determining the optimal MoO*_x_* thickness is paramount for the PSC performance. Figure S9, Supporting Information, shows the current density–voltage (*J*–*V*) curves of the SWNT–PI‐based PSCs with different MoO*_x_* film thicknesses under one‐sun illumination. The foldable PSCs with a 4‐nm‐thick MoO*_x_* film delivered the highest PCE. The cross‐sectional scanning electron microscopy (SEM) image of the device prepared using focused ion beam (FIB) milling shows the thicknesses of each layer in the PSCs (MoO*_x_* (4 nm)/PTAA (20 nm)/perovskite (450 nm)/C_60_ (20 nm)/BCP (6 nm)/Cu (50 nm)) (Figure 3b). As can be observed, the SWNTs are embedded in PI, forming a smooth surface. The energy diagram shows that the work function of the 4‐nm‐thick MoO*_x_*‐doped SWNT (≈5.1 eV) matches well with the valence band of PTAA (Figure 3c). This is supported by the highest *V*_OC_ achieved for the PSCs with a 4‐nm‐thick MoO*_x_* layer, which indicates efficient hole collection (Table S1, Supporting Information). Moreover, a high FF was achieved from the low series resistance (*R*_s_), which reveals that excessive MoO*_x_* deposition increases the *R*_s_ despite a decrease in the *R*_sheet_ of the CNT electrode. The foldable PSCs exhibited a PCE of 15.2% and an average PCE of 14.7% (**Table** **1** and Figure 3d). The devices did not exhibit hysteresis (Table S2 and Figure S10, Supporting Information). The obtained PCE may not be higher than those of PSCs fabricated on ITO/glass. This is because our devices are not only flexible but also foldable and can endure severe bending conditions. The obtained PCE is the record‐high efficiency among those of the reported flexible CNT‐based PSCs, and the mechanical stability is the highest among those of all the PSCs that appeared in the literature (**Table** **2**). The SWNT–PI‐based PSCs without a MoO*_x_* layer exhibited a PCE of 14.6% and an average PCE of 13.9% (Table 1 and Figure 3d). This uncovers the substantial *p*‐doping and the charge‐selective effect induced by MoO*_x_*. The enhancement in photovoltaic parameters was statistically analyzed (Figure S11, Supporting Information). The histogram shows a considerable increase in *V*_OC_, which implies improved hole‐collection owing to the well‐aligned energy levels of SWNTs and PTAA due to MoO*_x_* doping (Figure S11a, Supporting Information). However, the *J*_SC_ did not change significantly (Figure S11b, Supporting Information). The slight decrease in transmittance due to the presence of MoO*_x_* (Figure 1i) is considered to be compensated by the augmented current flow arising from the *p*‐doping. The integrated *J*_SC_ (17.6 mA cm⁻^2^) calculated from the external quantum efficiency (EQE) of the MoO*_x_*‐doped SWNT–PI‐based PSCs matches well with the *J*_SC_ calculated from the *J*–*V* curves (the integrated *J*_SC_ should be roughly 1% lower than the *J*_SC_ obtained via the *J*–*V* curve) (Figure S12, Supporting Information). The increases in *V*_OC_ and FF were determined via the electrochemical impedance spectroscopy (EIS) of the foldable PSCs with and without MoO*_x_* doping performed under different applied voltages (0, 0.2, 0.4, 0.6, and 0.8 V) (Figure S11a,c, Supporting Information). The resulting Nyquist plots are shown in Figure S13, Supporting Information, and the equivalent circuit is shown in Figure S13c, Supporting Information, which was used for the fitting of *R*_s_, charge‐transfer resistance (*R*_ct_), and recombination resistance (*R*_rec_) (Table S3, Supporting Information). The *R*_s_ and *R*_rec_ are plotted in Figures 3e and 3f, respectively. Under all applied voltages, the *R*_s_ values of the foldable PSCs with MoO*_x_* were on average 70% lower than those of the devices without MoO*_x_* (Figure 3e). This is ascribed to the decrease in *R*_sheet_ by approximately 80% upon MoO*_x_* doping (Figure 2d).^[^ ^39^, ^52^ ^]^ This is further confirmed by the Hall measurement of the SWNT–PI‐based conductors, which revealed an increase in carrier concentration, conductivity, and mobility upon MoO*_x_* doping (Table S4 and Figure S14, Supporting Information). Meanwhile, *R*_ct_ was obtained based on a high‐frequency response (Figure S13d, Supporting Information). Lower *R*_ct_ values indicate good charge transfer, but the *R*_ct_ values of the MoO*_x_*‐doped devices were only slightly higher than those of the non‐doped devices. We attribute this to the trade‐off between the energy level alignment and *R*_sheet_. The *R*_rec_ values of the MoO*_x_*‐doped devices were approximately twice those of the non‐doped devices under all applied voltages (Figure 3f). A high *R*_rec_ indicates a small electrical loss originating from non‐radiative recombination. This confirms a better energy level alignment between SWNTs and PTAA by MoO*_x_* doping, which contributes to the high FF and *V*_OC_ by increasing the shunt resistance (Figure S11a,11c, Supporting Information).

**Figure 3:**
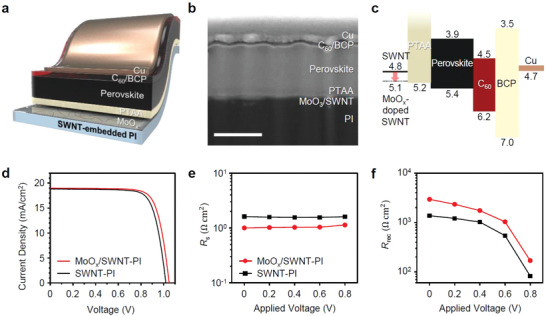
Ultrathin PSC employing the SWNT–PI conductor. a) Schematic illustration of the SWNT–PI‐based ultrathin foldable PSC, b) cross‐sectional SEM image of the devices (scale bar: 400 nm), c) energy level diagram of the devices, and d) *J*–*V* curves of the best‐performing SWNT–PI‐based devices with (red) and without (black) MoO*_x_*; e) series resistance (*R*_s_) and f) recombination resistance (*R*_rec_) of the devices with and without MoO*_x_* extracted from the Nyquist plots measured under different applied voltages.

**Table 1:** Photovoltaic parameters of the foldable PSCs with and without MoO*_x_* doping. The average values are obtained by performing measurements on 16 devices

		*V*_OC_ [V]	*J*_SC_ [mA cm^−2^]	FF [%]	PCE [%]
MoO*_x_*/SWNT–PI	Average	1.04 ± 0.01	18.7 ± 0.3	76.0 ± 1.2	14.7 ± 0.3
	Best	1.05	19.0	76.6	15.2
SWNT–PI	Average	1.02 ± 0.01	18.2 ± 0.6	74.9 ± 2.0	13.9 ± 0.5
	Best	1.02	18.8	76.2	14.6

**Table 2:** Performance comparison in terms of PCE and bending durability (test conditions and result) of our PSC with previously reported ITO‐free flexible PSCs

			Cyclic Flex Test				Ref.
Year	Electrode Type	Electrode Architecture	PCE [%]	Bending Radius [mm]	Bending cycles	PCE_final_/PCE_initial_ [%]	
2020	CNT	SWNT‐embedded PI	15.2	0.5	10 000	100	This work
2015	CNT	PET/SWNT/HNO_3_	5.38	10	N/A	85.5	^[^ ^30^ ^]^
2017	CNT	PEN/SWNT/MoO_3_	11	4	2,000	90	^[^ ^20^ ^]^
2020	Graphene	PET/EG‐Graphene	12.6	9	1,000	92.2	^[^ ^56^ ^]^
2018	Graphene	PDMS/Graphene	15	4	1,000	<80	^[^ ^57^ ^]^
2018	Graphene	PDMS/Graphene	18.2	4	5,000	43	^[^ ^58^ ^]^
2018	Graphene	PDMS/TFSA‐Graphene	17.5	4	5,000	≈35	^[^ ^58^ ^]^
2018	Graphene, CNT	Graphene, CSCNTs	11.9	4	2,000	84	^[^ ^59^ ^]^
2018	Graphene	PET/Graphene	13.94	4	1,000	≈92	^[^ ^59^ ^]^
2017	Graphene	PET/APTES/Graphene	17.9	4	100	>90	^[^ ^59^ ^]^
2016	Graphene	PEN/Graphene/MoO_3_	16.8	2	5,000	85	^[^ ^39^ ^]^
2019	PEDOT:PSS	PEDOT:PSS	19	3	5,000	≈80	^[^ ^39^ ^]^
2019	PEDOT:PSS	PEDOT:PSS	17.03	0.5	10,000	100	^[^ ^12^ ^]^
2019	PEDOT:PSS	PEDOT:PSS	20.25	2	5,000	97	^[^ ^60^ ^]^
2015	PEDOT:PSS	PEDOT:PSS	10.83	1	1,000	0.9	^[^ ^61^ ^]^
2019	AgNW	AgNW/PEDOT:PSS	15.06	5	1,000	80	^[^ ^62^ ^]^
2017	AgNW	a‐AZO/AgNW/AZO	11.23	12.5	400	94	^[^ ^63^ ^]^
2016	Metal grid	Ag grid/PET	14	5	5,000	95.4	^[^ ^64^ ^]^
2020	Metal grid	PI/Cu grid/Graphene	16.4	5	10,000	97	^[^ ^65^ ^]^
2019	Oxide/Metal/Oxide	TiO_2_/Ag/TiO_2_	13.00	1	1,000	97.6	^[^ ^18^ ^]^
2019	Oxide/Metal/Oxide	TiO_2_/Ag/TiO_2_	13.19	<1 (single folding)	50	85.3	^[^ ^16^ ^]^
				<1 (dual folding)	10	67.2	

The SWNT‐embedded PI‐based foldable PSCs demonstrated high reproducibility with no hysteresis. This is noteworthy since CNT‐laminated flexible PSCs shows a rather low reproducibility and high hysteresis.^[^ ^20^, ^30^, ^39^ ^]^ For better comparison, we fabricated flexible PSCs using the SWNT‐laminated PEN substrates (Figure S15a, Supporting Information). The cross‐sectional SEM image of the devices shows that the SWNTs on PET forms a rough interface between PTAA and the perovskite layer. Thus, the SWNT‐laminated PEN‐based devices exhibited a large hysteresis (Figure S15b, Supporting Information) and low reproducibility (Figure S15c, Supporting Information). This can be explained by considering the case of conventional PSCs. Hysteresis has been reported to occur when the bottom FTO electrode was not fully covered by a compact TiO_2_ layer and directly contacted the perovskite layer.^[^ ^53^ ^]^ Thus, the issues in the utilization of SWNT/PEN might be attributed to its direct contact with some protruded nanotubes and the perovskite layer. This is in stark contrast to the SWNT–PI interface, where the smooth morphology and absence of air traps prevent non‐radiative recombination (Figure S16, Supporting Information). Moreover, unlike the SWNT‐laminated PSCs, which required a thick PTAA layer on the SWNTs for surface roughness reduction, the SWNT‐embedded PI‐based PSCs needed a much thinner PTAA layer, which led to a much lower *R*_s_.

To qualify the developed PSCs as foldable devices (**Figure 4a**), harsh mechanical testing was conducted. Instead of the conventional bending test (bending radius, *R*_bending_ = 4 mm), we performed a folding test involving a folding radius (*R*_folding_) of 1 mm for the SWNT–PI‐based PSCs, SWNT‐laminated PEN‐based PSCs, and ITO‐deposited PEN‐based PSCs; the results are shown in Figure 4a. The ITO‐deposited PEN‐based PSCs exhibited a high PCE of 18.5% owing to the flat ITO surface and high transmittance of the PEN substrate (Table S5 and Figures S17a,b, Supporting Information). However, under a cyclic flex test with a *R*_bending_ of 4 mm, the PCE decreased continuously and diminished completely after 1 000 cycles (Figure 4b). This is not surprising since ITO develops cracks when bent at a *R*_bending_ of less than 5 mm.^[^ ^12^, ^13^ ^]^ Cracks were generated on the ITO layer after bending at *R*_bending_ = 4 mm, which led to abrupt performance degradation for the ITO/PEN‐based PSCs (Figure S17c, Supporting Information). The SWNT‐laminated PEN‐based PSCs could not withstand the same mechanical stress (Figure S18a, Supporting Information). Although the SWNT films easily withstood the bending test with a *R*_bending_ of 4 mm, the relatively poor adhesion between the SWNT films and PEN, as well as the thick PEN substrate (≈0.125 mm) caused the carbon electrodes to peel off during the cyclic flex test (Figure S18b, Supporting Information).^[^ ^32^ ^]^ The SWNT–PI‐based PSCs, on the other hand, withstood the bending test with a *R*_bending_ of 1 mm for more than 10 000 cycles with no drop in the PCE (Figure 4b). Furthermore, the devices endured the cyclic folding test with a R_folding_ of 0.5 mm without PCE reduction (Figure 4c). All the photovoltaic parameters, namely *V*_OC_, *J*_SC_, and FF, were retained after 10 000 folding cycles (Figure S19, Supporting Information), and no mechanical failure was observed in either the device or the ultrathin conductor, as determined from the cross‐sectional FIB‐SEM images obtained after the cyclic test (Figure S20, Supporting Information). We evaluated the mechanical stability of the SWNT–PI conductor‐based PSCs via a cyclic crumpling test. During the test, delamination and cracks occurred on the perovskite layer (Figures S21a,b, Supporting Information), and after 20 crumpling cycles, the electrical resistance of the SWNT–PI conductor increased by approximately 40% (Figure S21c, Supporting Information). Therefore, it is reasonable to assume that the percolated PI in the SWNT–PI composite affects the SWNT networks. The SWNT network might have disconnected in some areas under the large mechanical stress/strain induced by crumpling. An additional capping layer is necessary to adjust the mechanical neutral plane to impart mechanical stability against crumpling.^[^ ^12^ ^]^ The foldable PSCs demonstrated a maximum power point tracking output of 15.2% after the folding cycles (Figure 4d). We attribute the excellent mechanical resilience to the embedding of the SWNTs in PI as well as the ultra‐thinness of the SWNT–PI film (7 µm). Moreover, the devices were stable for over 25 days, which indicates that the lasting effect of MoO*_x_* doping in the SWNT–PI system is durable (Figure 1e).

**Figure 4:**
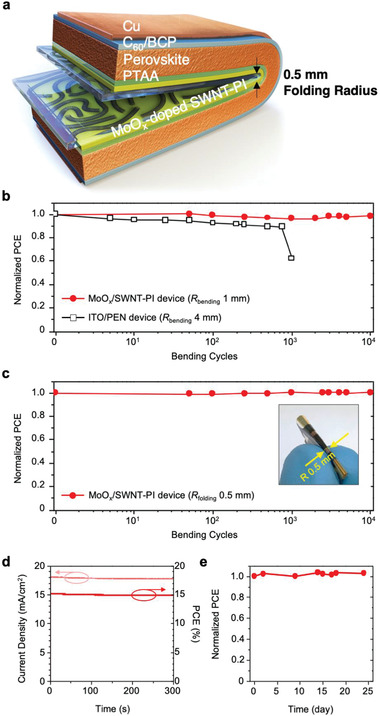
Folding durability of the SWNT–PI‐based ultrathin PSC. a) 3D illustration of the foldable PSCs fabricated in this work. b) Bending durability of the MoO*_x_*/SWNT–PI‐based device (*R*_bending_ 1 mm) and ITO/PEN‐based device (*R*_bending_ 4 mm) under different bending radii, c) folding durability of the MoO*_x_*/SWNT–PI‐based device (*R*_folding_ 0.5 mm), d) maximum power point tracking data of the MoO*_x_*/SWNT–PI device obtained under one‐sun illumination (100 mW cm^−2^), and e) long‐term stability of the device stored in a nitrogen‐filled glove box under dark condition at room temperature (≈25 °C).

In conclusion, foldable PSCs were realized using SWNT transparent electrodes for the first time to the best of our knowledge.^[^ ^17^, ^18^ ^]^ The higher requirements of foldable electronics, which mandates harsh mechanical conditions of 0.5 mm bending (or folding) radius, were met by implementing an ultrathin MoO*_x_*‐doped SWNT–PI matrix. The ultrathin SWNT–PI transparent conductor exhibited exceptional mechanical resilience, 7‐µm‐thinness, a smooth morphology, and a high DC‐to‐optical conductivity ratio. Along with the mechanical robustness of SWNTs, the SWNT–PI conductor demonstrated remarkable adhesion and ultra‐thinness, which are ideal for foldable electronic applications. Additionally, the percolation of PI through the SWNT network imparted a smooth surface to the SWNT–PI film and minimized recombination loss in the PSCs. Moreover, stable and strong MoO*_x_* doping was achieved by incorporating MoO_3_ during the curing process to induce anaerobic thermal *p*‐doping. This, together with the ultra‐thinness, resulted in an exceptionally high DC‐to‐optical conductivity ratio despite the infamous low transmittance of PI. The foldable PSCs showcased a power output of 15.2% with no hysteresis and could withstand over 10 000 folding cycles with a *R*_folding_ of 0.5 mm. The obtained results are some of the best among those reported thus far for flexible PSCs in terms of both efficiency and mechanical stability.

## Experimental Section

##### SWNT Synthesis

Randomly oriented SWNT networks with high purity and a long bundle length were synthesized via the aerosol chemical vapor deposition (CVD) method. The floating catalyst (aerosol) CVD was conducted in a scaled‐up reaction tube (150 mm diameter). The catalyst precursor was vaporized by passing ambient‐temperature CO through a cartridge filled with ferrocene powder. For stable SWNT growth, a controlled amount of CO_2_ was added with the carbon source (CO). The SWNTs were directly collected downstream of the reactor by filtering through a nitrocellulose or silver membrane filter (Millipore Corp., USA; HAWP, 0.45 µm pore diameter). The flow containing ferrocene vapor was then introduced into the high‐temperature zone of a ceramic tube reactor through a water‐cooled probe and mixed with additional CO. Ferrocene vapor was thermally decomposed in the gas phase of the aerosol CVD reactor at 880 °C. The CO gas was supplied at 4 L min^−1^ and decomposed on iron nanoparticles, resulting in the growth of SWNTs. The as‐synthesized SWNTs were collected via filtering with microporous filters at the downstream of the reactor. The transparency and *R*_sheet_ were controlled by varying the collection time.

##### SWNT–PI Ultrathin Conductor Fabrication

The collected square SWNT film (side: 1.2–1.8 cm) was mechanically deposited at the middle of a quartz glass substrate with an area of 2.5 × 2.5 cm^2^ via a dry‐transfer process (to prepare MoO*_x_*/SWNT–PI, MoO_3_ was deposited on the quartz substrate using thermal vacuum evaporation before transferring the SWNT film). Anhydrous ethanol (100 µL) was dropped on the substrate to densify the entangled SWNT network followed by drying at 70 °C for 1 min. The PI film was synthesized using a previously reported method.^[^ ^36^ ^]^ Equimolar amounts of 2,2‐bis[4‐(4‐aminophenoxy)pheny] hexafluoropropane (TCI) and 4,4′‐oxydiphthalic anhydride (TCI) were dissolved in anhydrous dimethylacetamide (DMAc, Sigma Aldrich) with a concentration of 19 wt% and mildly stirred for 12–15 h in a nitrogen‐filled glovebox. The viscous light‐yellowish precursor solution was spin‐coated on the SWNT/quartz substrate at 2 500 rpm (with an acceleration of 500 rpm s⁻^1^) for 60 s. During spin coating, the precursor solution percolates into SWNTs to form the SWNT–PI composite. The substrate was then dried at 90 °C for 5 min and transferred to a box furnace to cure PI. The sample was annealed at 200 °C for 20 min and 300 °C for 20 min to completely  cure the PI film. After cooling to room temperature, the SWNT‐embedded PI films were immersed in DI water where they peeled off from the quartz and floated in the water.

##### Solar Cell Fabrication

To fabricate PSCs on the ultrathin conductor, a polydimethylsiloxane (PDMS)‐coated quartz substrate was used as a rigid support and SWNT–PI was gently attached to it. Because of the adhesive nature of PDMS, the ultrathin conductor remained flat during the entire fabrication process.^[^ ^12^ ^]^ Then, PTAA (10 mg, Sigma Aldrich) was dissolved in 4‐isopropyl‐4′‐methyldiphenyliodonium tetrakis(pentafluorophenyl)borate (TPFB)‐added chlorobenzene (1 mL), which was prepared by dissolving TPFB (TCI) in chlorobenzene with a concentration of 1 mg mL⁻^1^. The solution was spin‐coated at 6 000 rpm for 30 s and then annealed at 100 °C for 10 min. Poly(9,9‐bis(3’‐(*N*,*N*‐dimethyl)‐*N*‐ethylammoinium‐propyl‐2,7‐fluorene)‐alt‐2,7‐(9,9‐dioctylfluorene))dibromide (PFN‐Br) was coated on PTAA as an interfacial compatibilizer.^[^ ^54^ ^]^ A solution of PFN‐Br (1‐Material) in anhydrous methanol (concentration: 0.4 mg mL⁻^1^) was spin‐coated at 4 000 rpm for 20 s. A perovskite film with a composition of MA_0.6_FA_0.4_PbI_2.9_Br_0.1_ was fabricated using a previously reported method.^[^ ^55^ ^]^ A perovskite precursor solution was prepared by dissolving PbI_2_ (461 mg, Sigma Aldrich), methylammonium iodide (MAI, 79.5 mg, Greatcell Solar), formamidinium iodide (FAI, 68.8 mg, Greatcell Solar), and MABr (11.2 mg, Greatcell Solar) in *N*‐dimethylformamide (DMF, 0.55 mL, Sigma Aldrich). A solution of urea (Sigma Aldrich) in dimethylsulfoxide (DMSO) (75 µL, urea concentration: 44.4 mg mL⁻^1^) was added to the aforementioned solution. The precursor solution was spin‐coated at 4 000 rpm for 20 s. Anhydrous diethyl ether (300–500 µL) was cast 5 s after the process was started. The intermediate perovskite turned black after annealing at 130 °C for 20 min. After spin coating, the samples were transferred into a nitrogen‐filled glovebox to complete the device. C_60_ (20 nm) and BCP (6 nm) were deposited by vacuum thermal evaporation at rates of 0.5 and 0.3 Å s^−1^, respectively. As a top electrode, Cu (50 nm) was deposited on the BCP film with an area of 0.60 cm × 0.15 cm (active area: 0.90 cm^2^). The spin‐coating processes were conducted at 25 °C and 10% relative humidity. The precursor solutions were filtered using a PTFE syringe filter with 0.45 µm pores before use. The vacuum thermal evaporation processes were conducted under a pressure of <5.0 × 10^−6^ Torr. After complete fabrication, the devices were detached from the PDMS/glass support for characterization.

##### Device and Film Characterization

The *J*–*V* curves of the PSCs were measured using a source meter (Keithley 2400, Tektronix) at a step voltage of 20 mV and delay time of 50 ms. The AM 1.5G one‐sun condition was realized using a solar simulator (Solar 3A Class, Oriel) with a KG‐5‐filtered standard silicon cell. During *J*–*V* measurements, the devices were covered with a photomask having an aperture area of 0.56 × 0.13 cm^2^. EIS was conducted using an electrochemical potentiostat (PGSTAT100N, Autolab). Cross‐sectional SEM images were obtained using a field‐emission scanning electron microscope equipped with an FIB (Auriga, Carl Zeiss). Optical transmittance spectra were measured using a UV–vis–NIR spectrophotometer (Cary 5000, Agilent Technologies). Surface topography images were obtained using an atomic force microscope (NX10, Park Systems). Bending tests were performed using a cyclic bending flexibility tester (ScienceTown) with a bending frequency of 1 Hz. The EQEs were measured using a quantum efficiency measurement system (IQE‐200B, Newport). XPS and UPS measurements were performed using a K‐Alpha Plus spectrometer (Thermo Fisher Scientific) with Al K*α* X‐ray radiation (1486.6 eV) and a Theta Probe spectrometer (Thermo Fisher Scientific) with HeI radiation (21.2 eV), respectively.

## Conflict of Interest

The authors declare no conflict of interest.

## Supporting information

Supporting Information
