# Supplementary material for: Foldable Perovskite Solar Cells Using Carbon Nanotube‐Embedded Ultrathin Polyimide Conductor
Source: Adv Sci (Weinh). 2021 Feb 8;8(7):2004092. doi: 10.1002/advs.202004092 (PMC8025023; doi:10.1002/advs.202004092)
Supplement: Supplementary file 1 — Supporting Information [file ADVS-8-2004092-s001.pdf]

© 2021 Wiley-VCH GmbH

ADVANCED SCIENCE
Open Access

Supporting Information

for *Adv. Sci.*, DOI: 10.1002/advs.202004092

Foldable Perovskite Solar Cells Using Carbon Nanotube-Embedded Ultrathin Polyimide Conductor

Jungjin Yoon, Unsoo Kim, Yongseok Yoo, Junseop Byeon, Seoung-Ki Lee, Jeong-Seok Nam, Kyusun Kim, Qiang Zhang, Esko I. Kauppinen, Shigeo Maruyama, Phillip Lee*, Il Jeon**

Supporting Information

Foldable Perovskite Solar Cells Using Carbon Nanotube-Embedded Ultrathin Polyimide Conductor

Jungjin Yoon, Unsoo Kim, Yongseok Yoo, Junseop Byeon, Seoung-Ki Lee, Jeong-Seok Nam, Kyusun Kim, Qiang Zhang, Esko I. Kauppinen, Shigeo Maruyama, Phillip Lee*, Il Jeon**

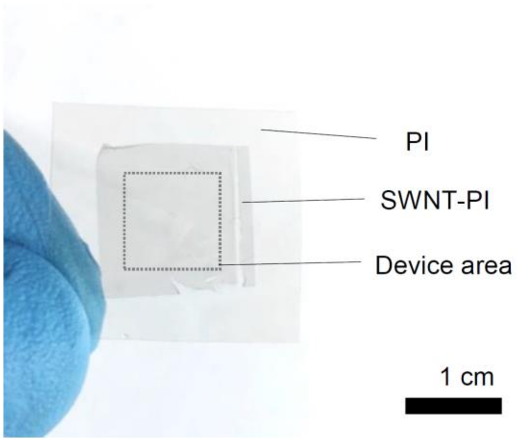

Figure S1. Digital photograph of the SWNT–PI conductor.

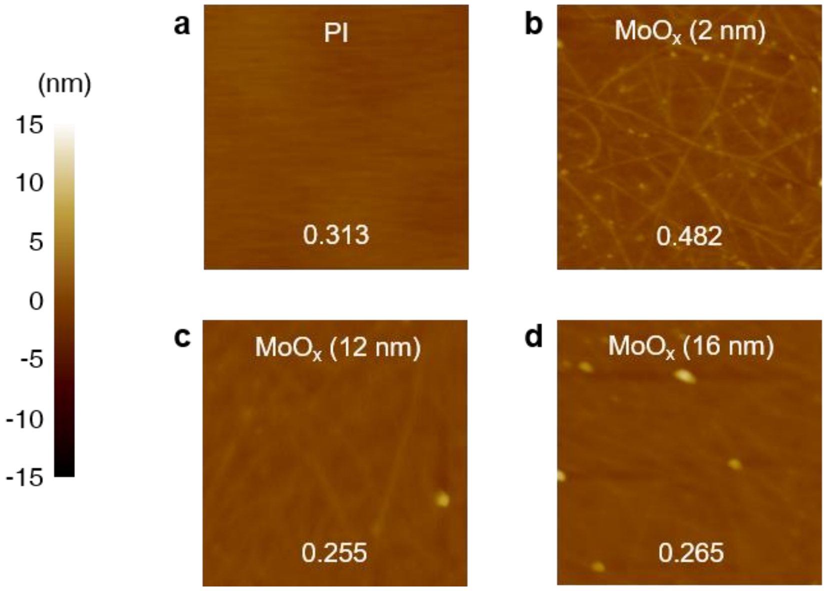

Figure S2. AFM topography images of (a) PI film, (b) MoO_x (2 nm)/SWNT–PI, (c) MoO_x (12 nm)/SWNT–PI, and (d) MoO_x (16 nm)/SWNT–PI.

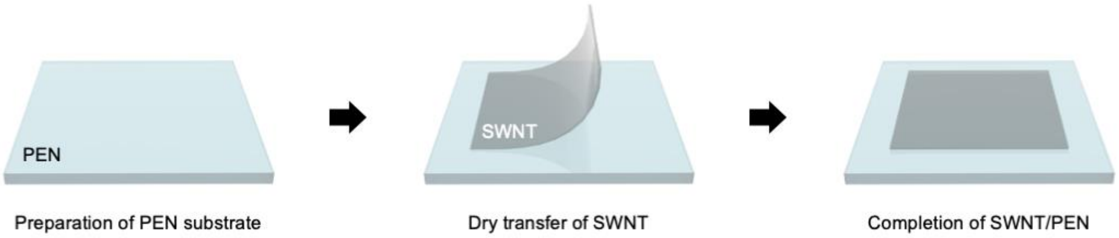

Figure S3. Schematic illustration of SWNT/PEN substrate preparation.

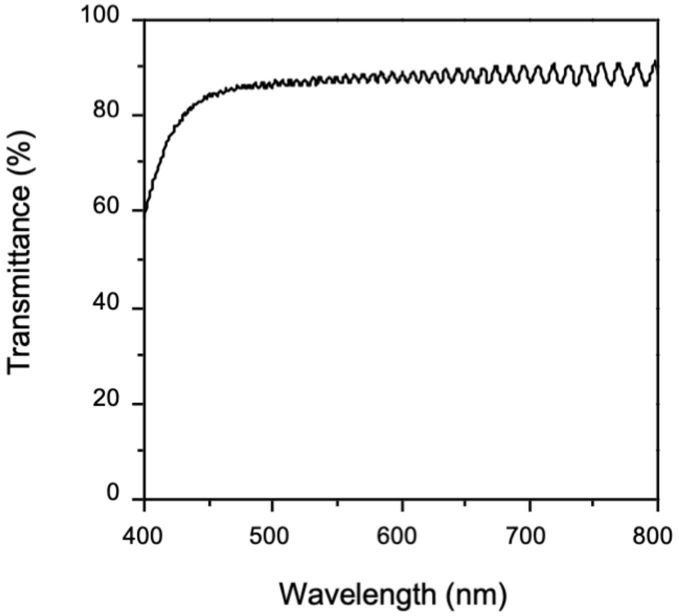

Figure S4. Optical transmittance spectrum of the PI film.

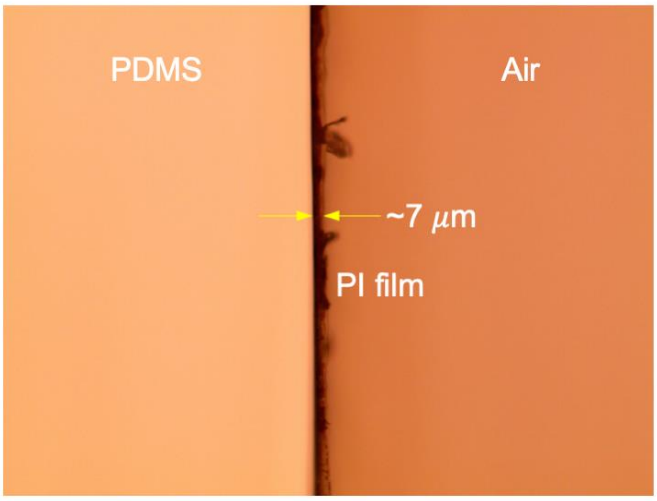

Figure S5. Cross-sectional optical microscope image of the PI film attached on a PDMS substrate.

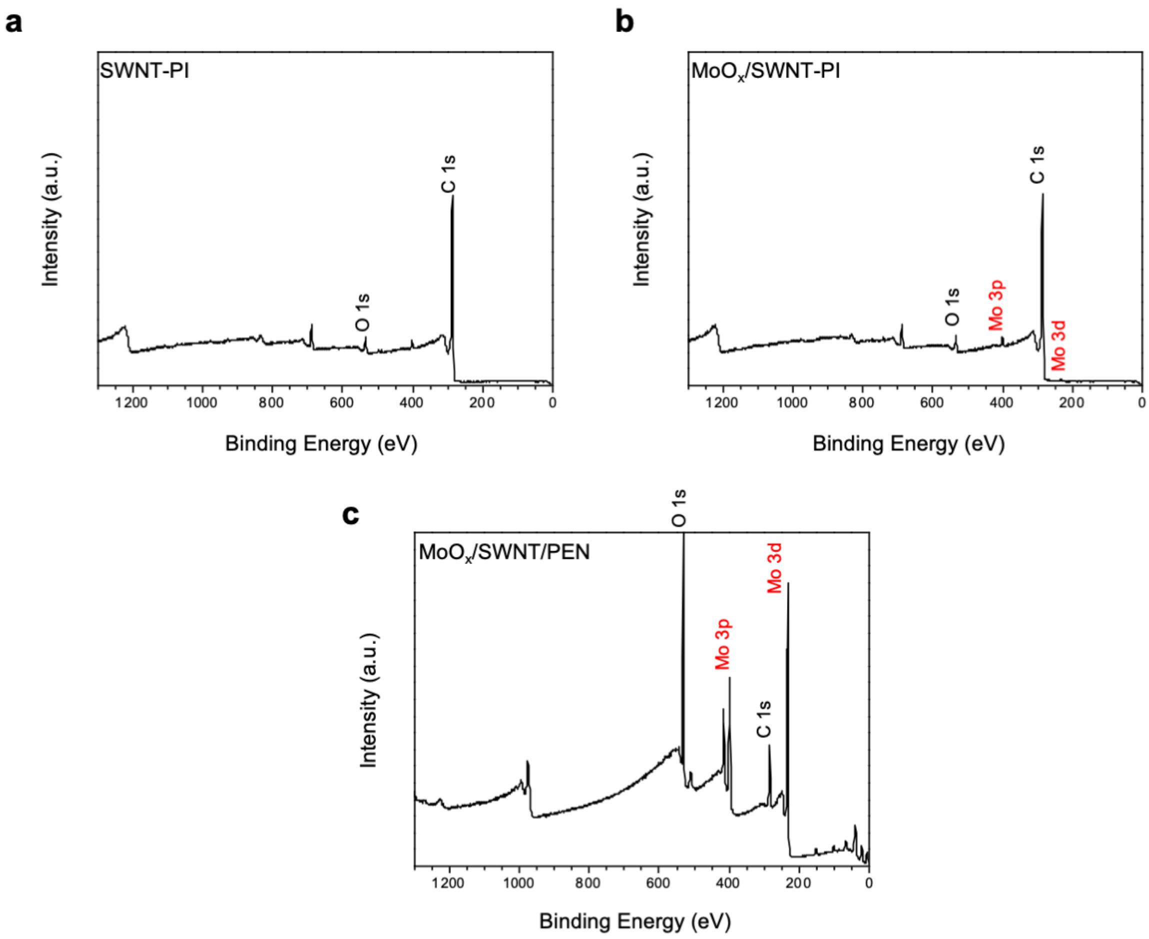

Figure S6. XPS spectra of different conductor configurations (a) SWNT–PI, (b) MoO_x/SWNT–PI, and (c) MoO_x/SWNT/PEN (peak positions of O 1s, C 1s, Mo 3p, and Mo 3d are indicated)

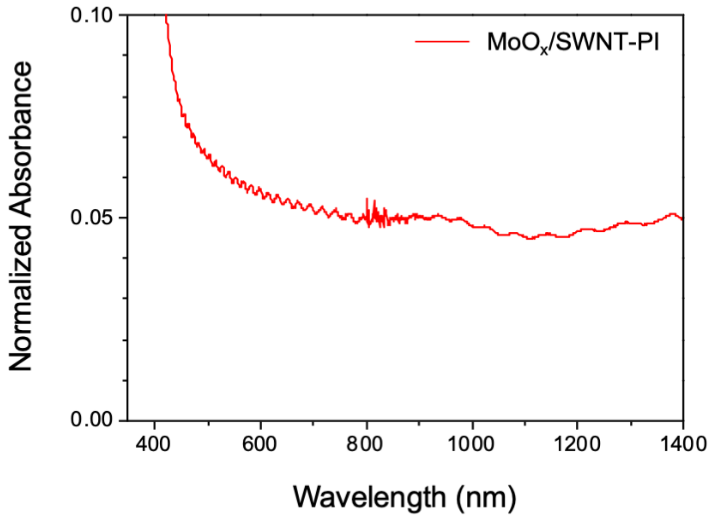

Figure S7. Normalized absorbance spectrum of the ultrathin conductor. Fluctuations can be seen over the entire wavelength range due to optical interference of the thin substrate.

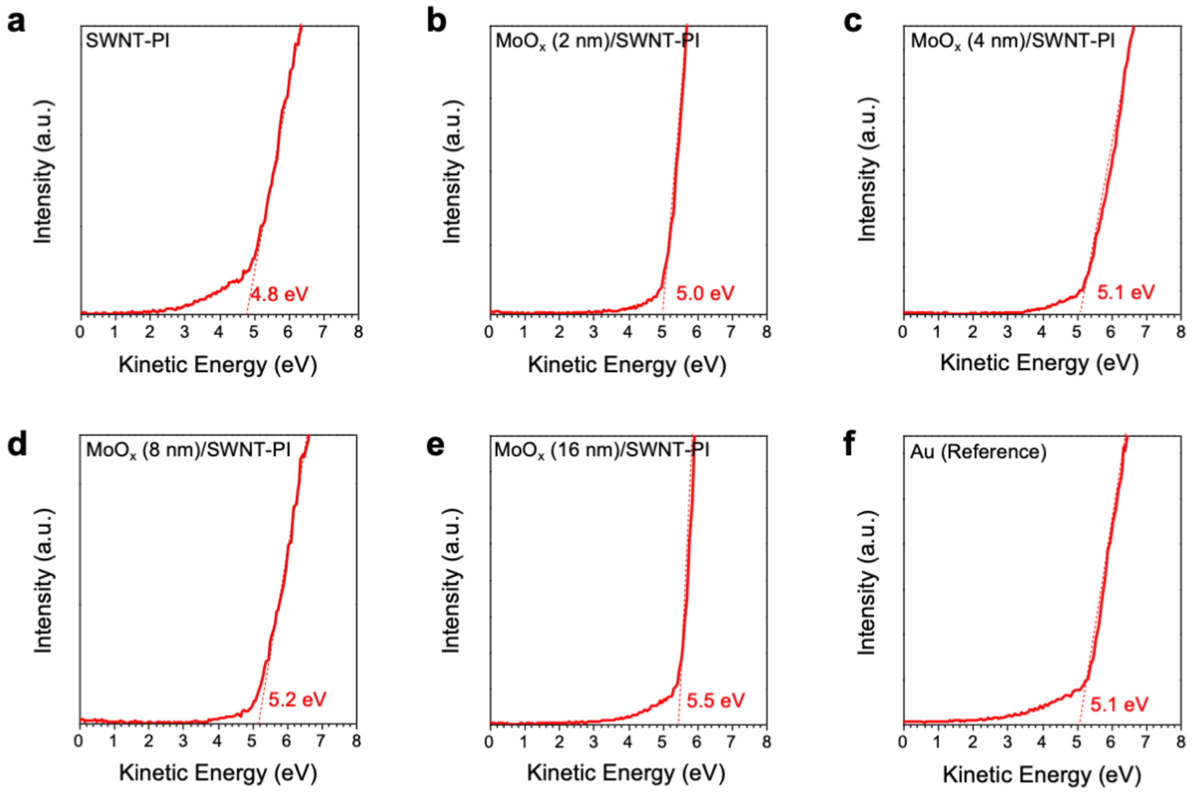

Figure S8. UPS spectra (a) SWNT–PI, (b) MoO_x (2 nm)/SWNT–PI, (c) MoO_x (4 nm)/SWNT–PI, (d) MoO_x (8 nm)/SWNT–PI, (e) MoO_x (16 nm)/SWNT–PI, and (f) reference Au film.

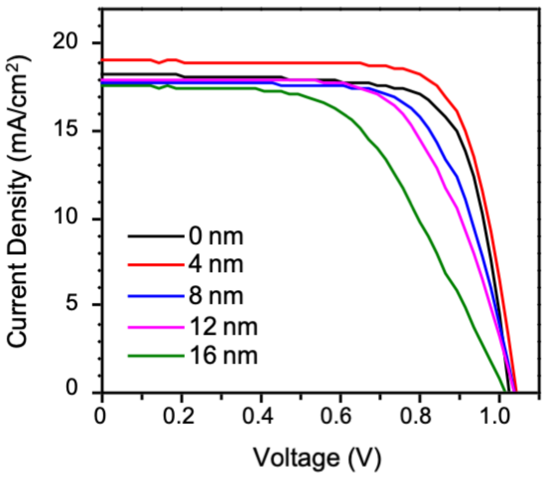

Figure S9. *J*–*V* curves of perovskite solar cells employing MoO_x/SWNT–PI with different MoO_x thicknesses

Table S1. Summary of photovoltaic parameters of the MoO_x/SWNT–PI-based devices corresponding to Figure S9.

MoO_x thickness (nm)	*V_{OC} [V]*	*J_{SC} [mA cm^{-2}]*	*FF [%]*	*PCE [%]*	*Sheet resistance of SWNT–PI [Ω sq.$^{-1}$]*
0	1.03	18.2	74.3	13.9	100.8
4	1.05	19.0	74.9	14.9	82.0
8	1.04	17.8	69.0	12.8	78.1
12	1.04	18.0	64.7	12.1	49.0
16	1.02	17.6	55.0	9.8	40.3

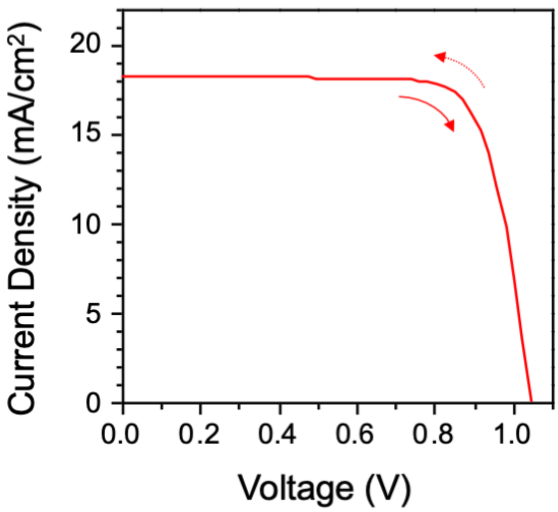

Figure S10. *J*–*V* curves of a foldable PSC employing a SWNT–PI conductor with 4-nm-thick MoO_x layer obtained under different voltage sweep directions.

Table S2. Photovoltaic parameters of an ultrathin PSC measured under different voltage sweep directions corresponding to Figure S10.

Scan Direction	***V_{OC} [V]***	***J_{SC} [mA cm^{-2}]***	***FF [%]***	***PCE [%]***
Reverse	1.04	18.3	77.6	14.9
Forward	1.04	18.3	77.3	14.8

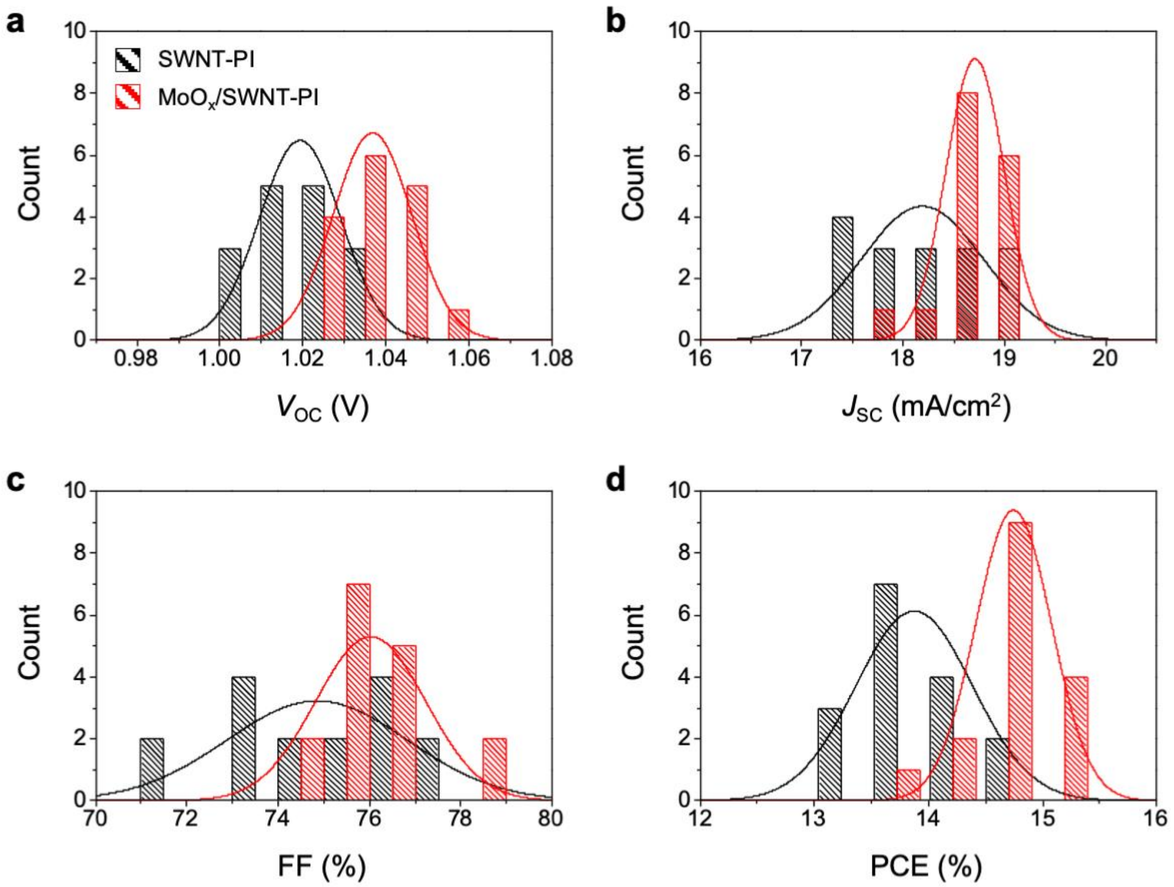

Figure S11. Histograms of photovoltaic parameters: (a) V_{OC}, (b) J_{SC}, (c) FF, and (d) PCE

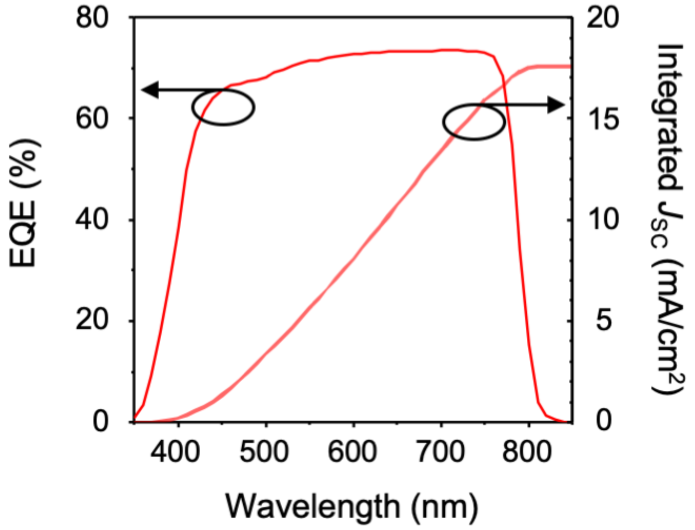

Figure S12. EQE data of the PSC fabricated on MoO_x/SWNT–PI (red line, y_1-axis). J_{SC} calculated from the EQE curve is 17.6 mA cm^{-2} (pink line y_2-axis).

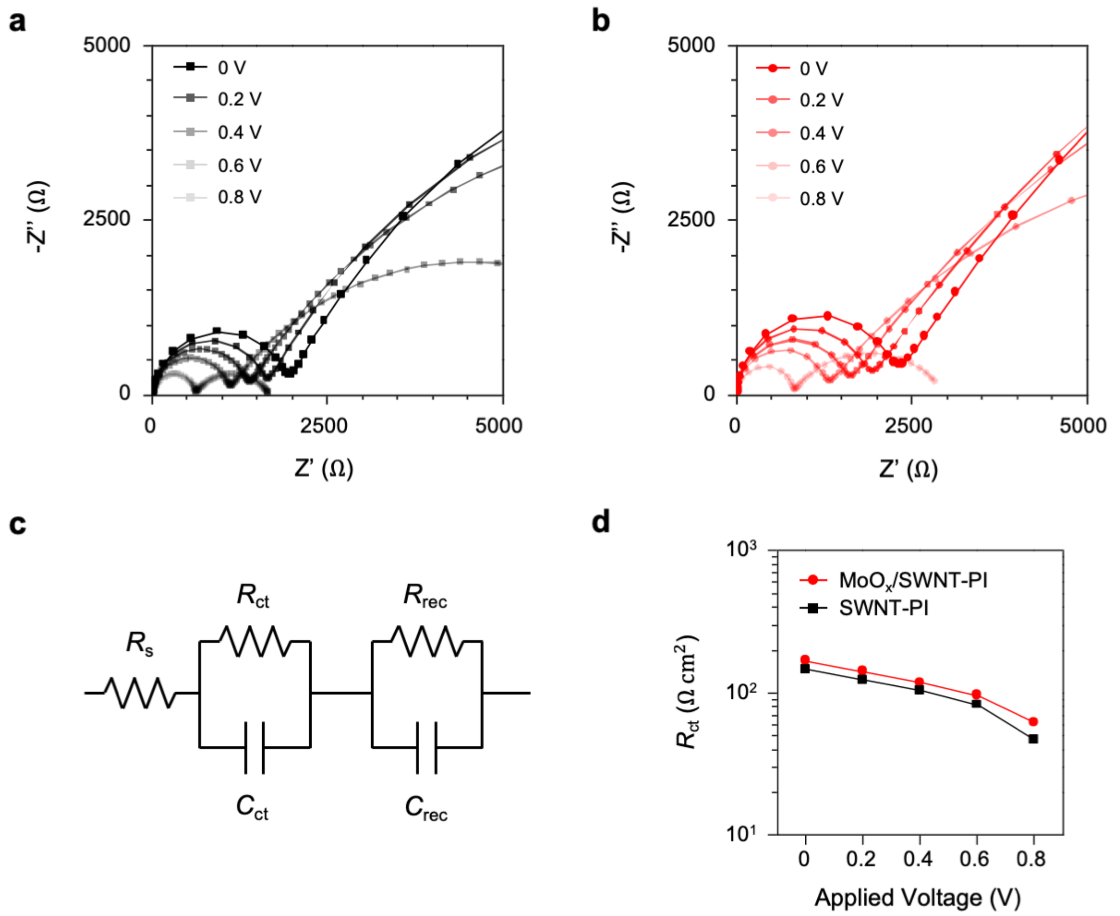

Figure S13. EIS analysis results. Nyquist plots of the devices (a) without and (b) with MoO_x doping obtained under different applied voltage conditions, (c) an equivalent circuit to fit the plots, and (d) charge transfer resistance (R_{ct}) of the devices.

Table S3. Fitted parameters extracted from the Nyquist plots obtained by EIS analysis corresponding to Figure S13.

Applied Voltage (V)	R_s [Ω cm^2]		R_{ct} [Ω cm^2]		R_{rec} [Ω cm^2]	
	SWNT–PI	MoO_x/SWNT–PI	SWNT–PI	MoO_x/SWNT–PI	SWNT–PI	MoO_x/SWNT–PI
0	1.61	1.00	146.48	168.48	1371.86	2945.36
0.2	1.57	1.02	123.63	141.18	1210.01	2329.70
0.4	1.56	1.02	103.66	118.01	1020.08	1739.56
0.6	1.56	1.04	82.91	96.17	541.24	1030.93
0.8	1.60	1.13	47.01	61.94	82.21	169.03

Table S4. Hall measurement data for SWNT-PI conductors

	Bulk concentration [cm^{-3}]	*Conductivity [S cm^{-1}]*	*Mobility [cm^2 V^{-1} s^{-1}]*
SWNT	8.21 X 10^{20}	101.6863	1.002483
MoO_x-doped SWNT	2.46 X 10^{21}	191.9417	1.254625

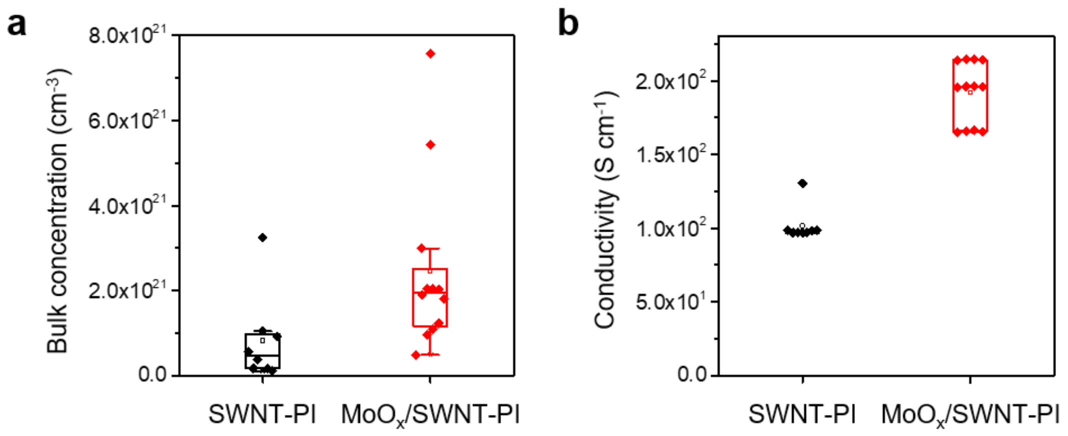

Figure S14. Box charts for (a) bulk concentration and (b) conductivity of the SWNT-PI conductors without and with MoO_x doping

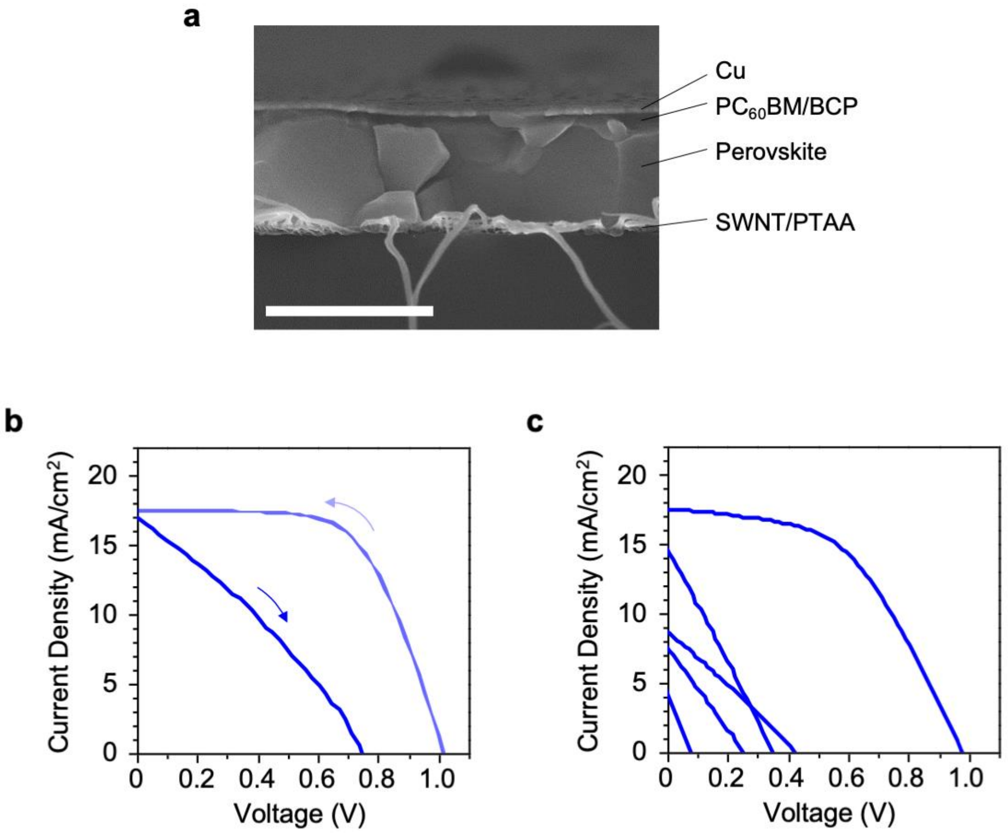

Figure S15. (a) Cross-sectional SEM image of a device fabricated on SWNT/PEN (scale bar: 1 µm), (b) *J*–*V* curves of the same device measured with different voltage sweep directions, and (c) *J*–*V* curves of the SWNT/PEN-based devices showing low reproducibility.

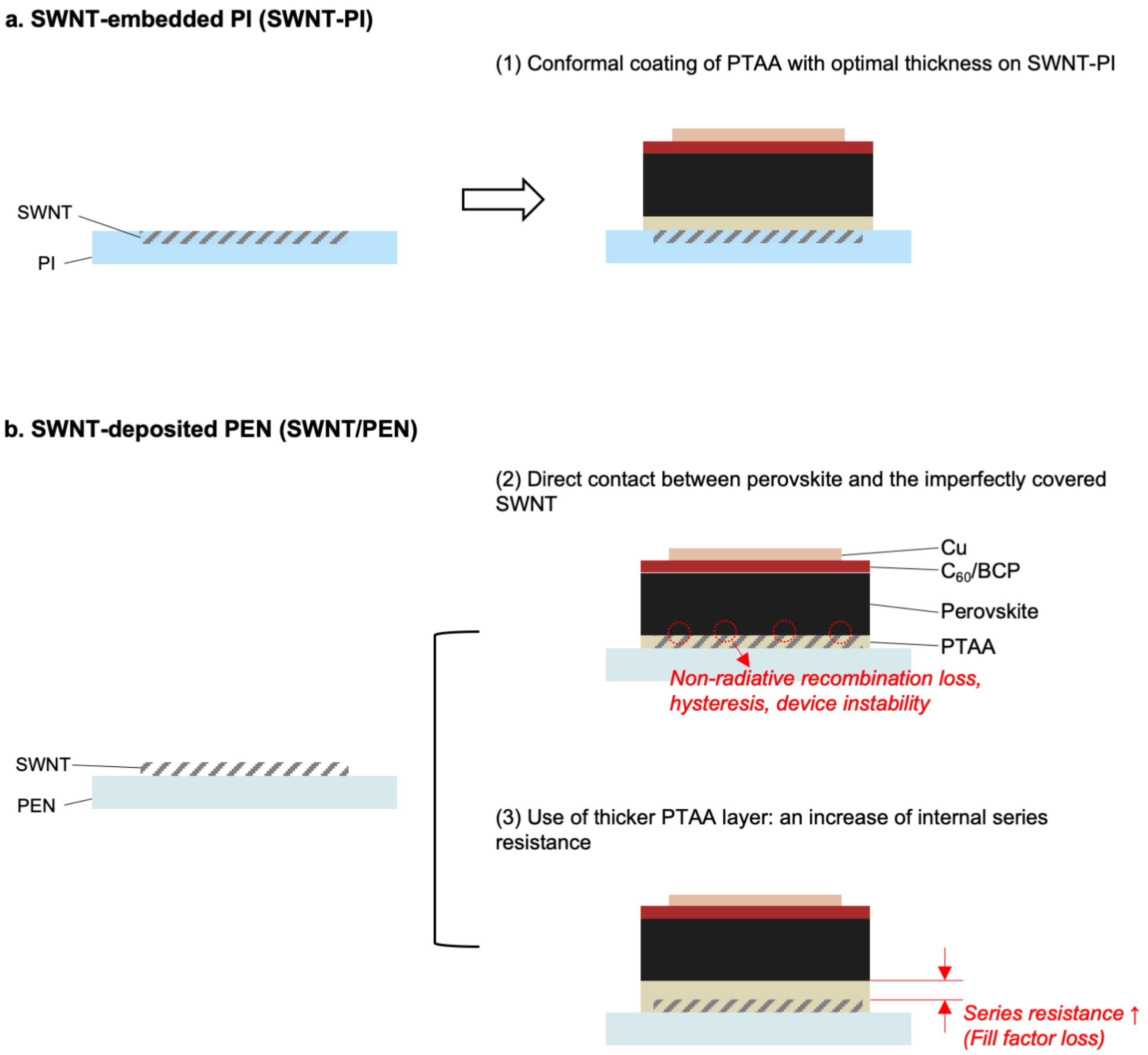

Figure S16. (a) Schematic illustration of the device based on SWNT-embedded PI, and (b) an explanatory depiction of the performance loss of the devices based on SWNT/PEN.

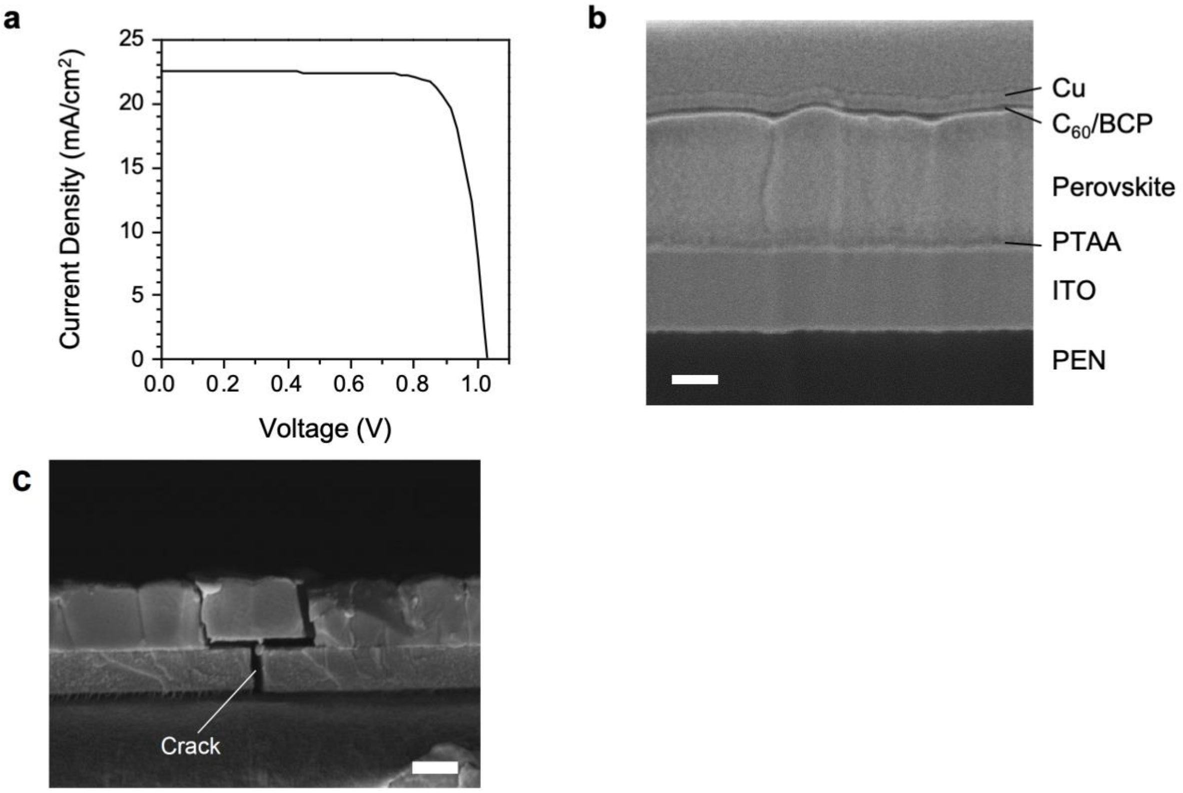

Figure S17. (a) *J–V* curve of the ITO-based flexible PSC, (b) a cross-sectional FIB-SEM image of the ITO-based device (scale bar: 200 nm), and (c) cross-sectional SEM image of the device after 1,000 bending cycles with $R_{bending}$ = 4 mm (scale bar: 400 nm)

Table S5. Photovoltaic parameters of the ITO-based flexible PSC corresponding to Figure S17a.

V_{OC} [V]	J_{SC} [mA cm^{-2}]	FF [%]	PCE [%]
1.03	22.5	79.8	18.5

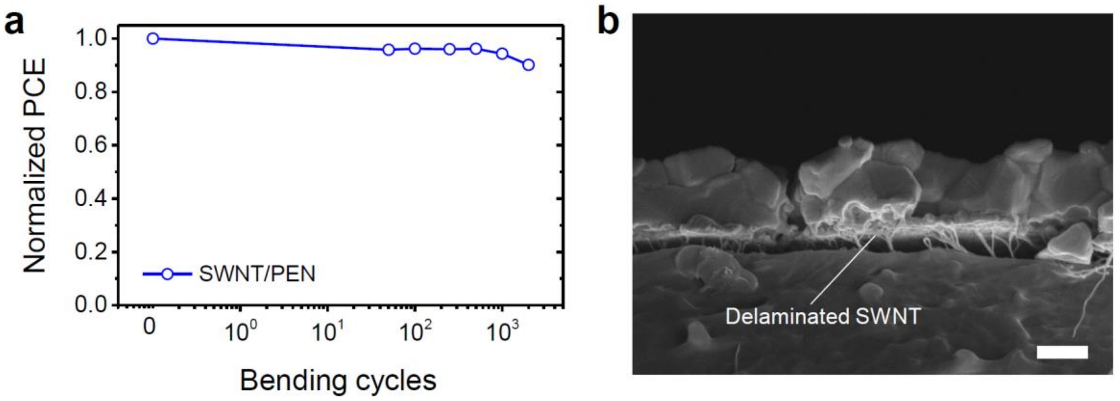

Figure S18. (a) Normalized PCE of the flexible PSC based on SWNT/PEN as a function of bending cycles with $R_{bending}$ = 4 mm, and (b) cross-sectional SEM image of the device after 2,000 cycles under the same test conditions (scale bar: 400 nm).

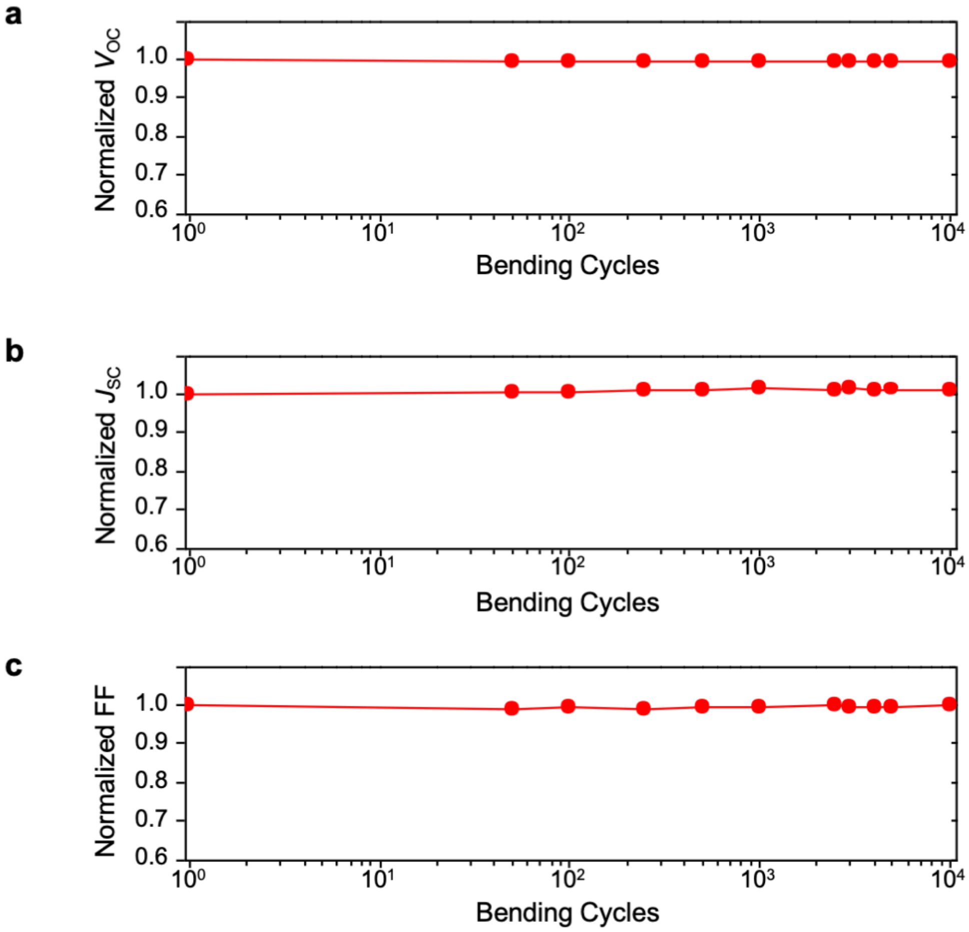

Figure S19. Normalized photovoltaic parameters of the foldable PSCs employing the MoO_x/SWNT–PI conductor as a function of folding cycles with a $R_{folding}$ of 0.5 mm: (a) V_{OC}, (b) J_{SC}, and (c) FF.

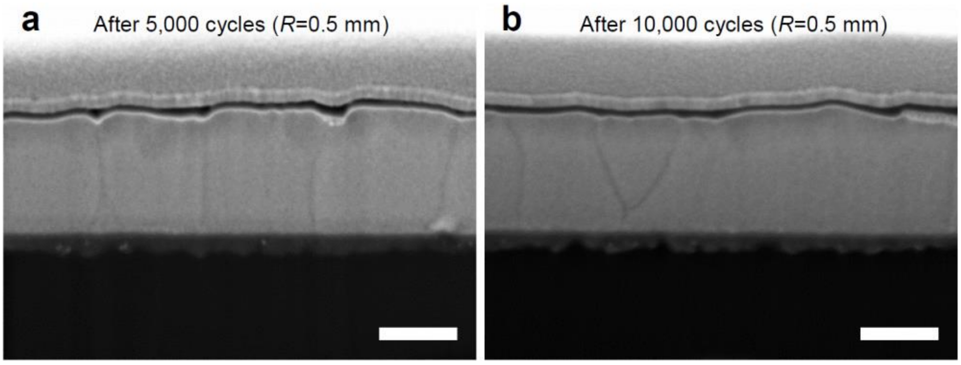

Figure S20. FIB-assisted cross-sectional SEM images of the SWNT–PI-based devices after (a) 5,000 cycles, and (b) 10,000 cycles with a folding radius of 0.5 mm (scale bars: 400 nm).

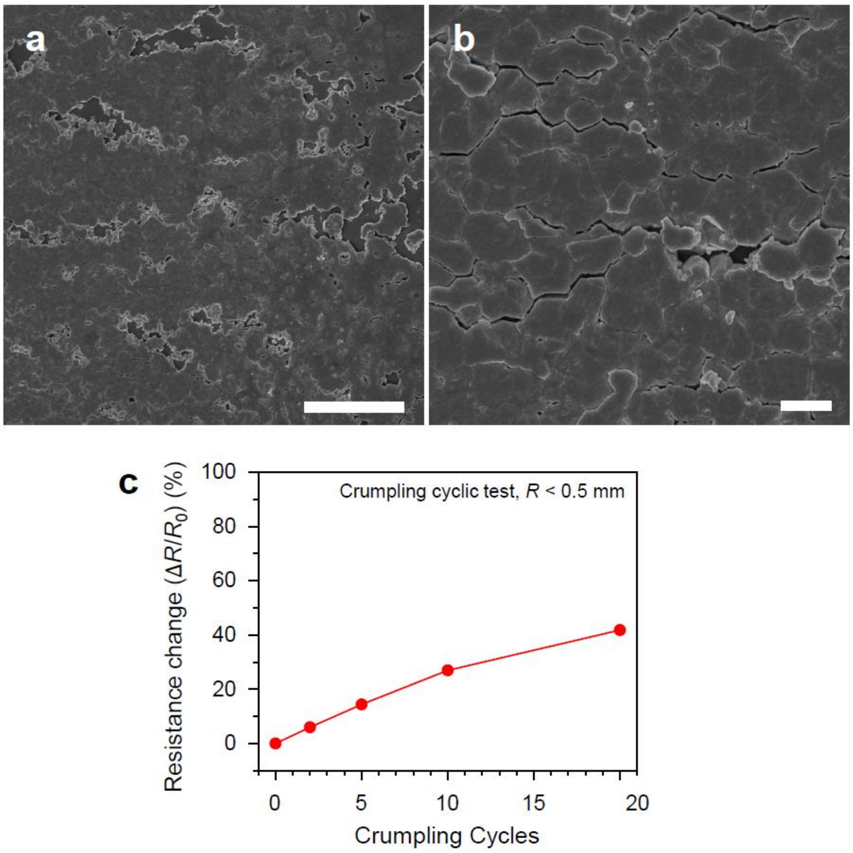

Figure S21. Top-view SEM images of the perovskite film coated on the SWNT–PI conductor after the crumpling test: (a) low-magnification image (scale bar: 8 μm) and (b) high-magnification image (scale bar: 1 μm). (c) Change in electrical resistance of the SWNT–PI conductor over crumpling cycles.
